# There is no market for new antibiotics: this allows an open approach to research and development

**DOI:** 10.12688/wellcomeopenres.16847.1

**Published:** 2021-06-11

**Authors:** Dana M. Klug, Fahima I. M. Idiris, Mark A. T. Blaskovich, Frank von Delft, Christopher G. Dowson, Claas Kirchhelle, Adam P. Roberts, Andrew C. Singer, Matthew H. Todd

**Affiliations:** 1 School of Pharmacy, University College London, London, WC1N 1AX, UK; 2 Centre for Superbug Solutions, Institute for Molecular Bioscience, The University of Queensland, Lucia, Queensland, 4072, Australia; 3 Centre for Medicines Discovery, The University of Oxford, Oxford, OX3 7DQ, UK; 4 Diamond Light Source Ltd, Didcot, OX11 0QX, UK; 5 Department of Biochemistry, University of Johannesburg, Auckland Park, 2006, South Africa; 6 School of Life Sciences, University of Warwick, Coventry, CV4 7AL, UK; 7 School of History, University College Dublin, Dublin, Ireland; 8 Department of Tropical Disease Biology, Liverpool School of Tropical Medicine, Liverpool, L3 5QA, UK; 9 UK Centre for Ecology & Hydrology, Wallingford, OX10 8BB, UK

**Keywords:** antibiotics, AMR, policy, economics, open science, incentives, drug discovery, drug development

## Abstract

There is an increasingly urgent need for new antibiotics, yet there is a significant and persistent economic problem when it comes to developing such medicines. The problem stems from the perceived need for a “market” to drive commercial antibiotic development. In this article, we explore abandoning the market as a prerequisite for successful antibiotic research and development. Once one stops trying to fix a market model that has stopped functioning, one is free to carry out research and development (R&D) in ways that are more openly collaborative, a mechanism that has been demonstrably effective for the R&D underpinning the response to the COVID pandemic. New “open source” research models have great potential for the development of medicines for areas of public health where the traditional profit-driven model struggles to deliver. New financial initiatives, including major push/pull incentives, aimed at fixing the broken antibiotics market provide one possible means for funding an openly collaborative approach to drug development. We argue that now is therefore the time to evaluate, at scale, whether such methods can deliver new medicines through to patients, in a timely manner.

## Disclaimer

The views expressed in this article are those of the authors. Publication in Wellcome Open Research does not imply endorsement by Wellcome.

## 1) We need new antibiotics and access to antibiotics

Antimicrobial resistance (AMR) is a serious threat to global public health because it leads to the spread of bacteria that cannot be eliminated or controlled with existing medicines ^1, 2^. We have been extensively warned that we are entering a post-antibiotic era ^3^ as antibiotic-resistant bacteria now cause an estimated 25,000 deaths in Europe alone every year ^4– 6^. Nevertheless, in 2015 more people still died from lack of access to otherwise effective antibiotics than antibiotic resistance ^7^. As of 2019, there were 43 antibiotics and combinations which contain a new therapeutic entity in clinical or preclinical development for the treatment of serious bacterial infections, but only 15 of these antibiotics have the potential to treat pathogens listed as priorities by the World Health Organisation (WHO) ^8^ and there is particular urgency regarding the lack of new treatments for gram-negative pathogens. As the success rate of the drug development pipeline remains low, there is an urgent need for novel antibiotics to circumvent the threat from AMR, and our ability to treat common infections ^9^.

## 2) There is an economic problem in developing antibiotics

The central problem of the empty antibiotic pipeline is not scientific but economic. Between the 1930s and 1960s, significant antibiotic innovation resulted from large-scale public and private screening programs and targeted drug development, initially driven by the lack of effective antibacterial treatments ^10, 11^. By the early 1980s, private investment in antimicrobial research ebbed as a result of generational change within pharmaceutical companies and a broader reorientation of private research and development (R&D) towards more focused investment in expensive yet lucrative noncommunicable (e.g., cancer and lifestyle) medications ^12, 13^ as well as a change in a perception of need, arising from the effectiveness of existing antibiotics ^14^. The decline in private investment was exacerbated by the parallel closure of formerly successful public R&D efforts, as a result of the contemporary political emphasis on privatization and marketplace-oriented research.

The disappearance of substantial investment in functionally new antibiotic classes did not mean that scientific research on new antibiotics had reached an impasse ^15, 16^. During the 1980s and 1990s, smaller biotech start-ups, publicly funded researchers, and researchers within larger companies continued to employ classic screening and new molecular approaches to find and develop promising compounds ^17, 18^. The problem was that there was no longer sufficient public or private investment for these compounds to move beyond early stages of pre-clinical development or clinical trialling ^19– 23^. Already diagnosed in the mid-1990s, the described disconnect between scientific potential and lack of substantial development support from industry continues to this day. 

Since 2010, the United States Food and Drug Administration (FDA) has approved approximately 20 new antimicrobials ^24^. However, from a classic economics perspective, antibiotics remain unable to compete with the profitability of blockbuster drugs. The profitability of the broader antibiotic market is constrained by the widespread availability of competitive cheap generic compounds (“crowding out” newer, more expensive medicines), the ‘short course’ treatment afforded by the rapid ‘chemical surgery’ antibiotics provide, and current reimbursement practices ^1, 25^. Potential market size for new compounds is further constrained by stewardship concerns, and profitability is lowered further by the cost of meeting regulatory requirements and the low prices of currently marketed antibiotics. There is accordingly a significantly delayed economic return on the investment that created the drug, in many cases destroying the traditional economic incentive for drug development (this situation is commonly referred to as the “fire extinguisher problem,” referring to an object that is important to have, but not to use) ^26, 27^. Investing in new antibiotics is considered high risk, driving companies to decide to move away from antimicrobial research ^28, 29^; high profile examples from 2016–2019 include Novartis, Sanofi, AstraZeneca, and GSK ^30– 33^.

Small and medium-sized enterprises (SMEs) are major contributors to preclinical development globally, accounting for 81% of antibacterial programmes ^34, 35^. Although a driving force in innovation, SMEs are struggling to finance their efforts ^1, 28, 29^. The recent bankruptcies of Achaogen, Tetraphase Pharmaceuticals, Aradigm, and Melinta Therapeutics – all SME biotechs which recently gained FDA approval for novel antibiotics *and which were actually selling them* – highlights the need for new political and economic models for antimicrobial research and development ^1, 23, 29, 36, 37^.

## 3) Is there an antibiotics market?

One must inevitably ask: *is there a viable market for new antibiotics?* Are attempts to revive and use traditional systems of market lure to combat AMR going to work?

If the answers are ‘no’, and we abandon the notion that a profit is *required*, then we open up different ways of conceiving the development of new medicines: ones that are not required to align with the rules designed to deliver economic return. If we also abandon the idea of a ‘market’ for new antibiotics, acknowledging the market failures of the current approach ^38^ and reframe antibiotics as public goods that keep established health-care infrastructure running and serve as a foundation for developing health-care systems, then we can develop an orthogonal development model that replaces, or at least sits alongside, the current market-led, for-profit development approach. On the one hand, public funding must play a key role in future efforts, but on the other hand new development and reimbursement mechanisms can be conceived and trialled alongside existing public stimuli programs for private initiatives.

Despite an abundance of successful real-world models of public pharmaceutical development, Western state and non-governmental funders have, since the 1980s, almost exclusively adopted neoliberal economic thinking, which demands a dominant role for the private sector in the full translation to clinical practice, and which simply dismisses the public sector as an option. However, this is a political emphasis on market-based solutions, and glosses over ample real-world evidence for successful public R&D projects that resulted in the rollout of transformative pharmaceutical interventions - including penicillin, which was famously not patented ^10^. (The current COVID19 pandemic may well provide further starting points for new development and distribution models due to the major challenge of rolling out vaccines globally.) This imbalance is being addressed through the exploration of new development solutions targeting early-stage scientific initiatives ( Section 4) and economic models ( Section 5).

## 4) There are initiatives aimed at finding new starting points and monitoring activity

There are already several initiatives outside the pharma industry aimed at facilitating the early-stage discovery of new antibiotics. Agencies such as the Directorate-General for Research and Innovation, European Commission (DG-RTD), the US National Institutes of Health (NIH) ^39^, and the UK National Institute for Health Research (NIHR) have recognized the need for funding basic research grants and fellowships and have launched internal initiatives ^40, 41^. Public-private partnerships such as CARB-X ^42, 44^ and the Innovative Medicines Initiative’s New Drugs for Bad Bugs (ND4BB) ^45, 46^ aim to distribute risk among public and private entities while providing platforms for collaboration and resource-sharing. Other initiatives, such as the Pew Trust’s SPARK platform ^47– 49^, which provides an open, curated database of antibacterial research, and the Community of Open Antimicrobial Drug Discovery (CO-ADD) ^50– 53^, a free screening service against high-priority ESKAPE bacteria and fungal pathogens, encourage participation of new researchers, and the open sharing of information and resources ^54^. The Revive initiative from The Global Antibiotic Research and Development Partnership (GARDP), a joint initiative between the World Health Organization and Drugs for Neglected Diseases initiative (DNDi), aims to ensure that knowledge gained in previous and ongoing antimicrobial campaigns is shared throughout the antibiotic drug discovery community, and that new projects can benefit from the experience of those best placed to advise ^55, 56^. In addition, the Access to Medicines Foundation has recently published the 2020 AMR Benchmark evaluating how 30 pharmaceutical companies, all of which voluntarily provide data for the scoring evaluation exercise, are responding to the global threat of AMR ^57^. Combined, these initiatives provide a framework for funding, data sharing and information mining, and research support in service of accelerating the discovery of new antibacterial agents. Despite encouraging the community towards greater collaborative efforts, the feature behind many, perhaps all, of these initiatives is that they assume that ultimately market forces will still be needed to bring developments to the clinic.

## 5) Some proposed solutions to the economic challenge

State and non-governmental actors have started to respond to the fundamental economic challenge of market-led antibiotic R&D with several proposed remedies ^58, 59^. The final report of the Review on Antimicrobial Resistance suggested that substantial push (direct funding, pre-approval) and pull (incentive, post-approval) mechanisms would likely be needed ^5, 60, 61^. Regulatory agencies such as the European Medicines Agency and the US FDA have offered incentives designed to increase the market exclusivity period for new antibiotics, thereby increasing their profitability ^40^. Significant push incentives are being offered through investment in early stage projects by CARB-X and the Novo REPAIR Fund ^62, 63^. The pharmaceutical industry has recently come together to form the AMR Action Fund ^64^, an unprecedented push collaboration with the World Health Organisation (WHO), The European Investment Bank (EIB), and the Wellcome Trust to invest nearly $1bn focussing on the later stages of development and which aims to bring two to four new antibiotics to patients by 2030. These very welcome investments of resources are nevertheless just the beginning of the commitment that is likely to be needed ^65^. Note added in proof: a recently-published analysis (REF) suggests that the discounted net present value of a new antibiotic is $240M, considerably below the probable development costs, and that the US market accounts for >80% of sales.

Other solutions to the antibiotics market involve pull incentives that attempt to provide a guaranteed income stream for companies that successfully bring molecules to market, the so-called ‘delinked market entry rewards’. These rewards attempt to fix one of the most serious of the market failures, that companies selling approved, novel antibiotics can nevertheless still go bankrupt. Recently, the UK has announced that it will trial a ‘subscription model’ of paying for antibiotics, in which health providers will pay for future access to antibiotics kept in reserve, regardless of the level of use (i.e., ‘delinkage’) ^66– 68^. Similarly, the Swedish government has commissioned the Public Health Agency of Sweden (PHAS) to propose and pilot new models for keeping approved antibiotics available on the Swedish market, while contending with the overuse that can lead to resistance ^69^. The PASTEUR Act, currently proceeding through governmental approval in the US, aims for a similar outcome ^70, 71^. Such schemes rely on other governments stepping up with similar levels of (proportional) commitment ^72^.

Reimbursement models are challenging when considering new, breakthrough medicines, where the cost of that medicine needs to be weighed against the lower cost of one that is comparatively less effective. If the ‘cost’ of a drug is interpreted simply, there may be financial pressure for new, expensive medicines to be prescribed less frequently than cheaper, less effective medicines, purely to reduce the outlay on the drug itself. Of course, this ignores the very significant costs associated with ongoing patient care (e.g., non-surgical hospital admission and nosocomial infection). In late 2019, the USA Centers for Medicare & Medicaid Services announced it was changing the way hospitals are reimbursed for antibiotics and treatment of antimicrobial resistance, improving the ability for resistant infections to be classified for higher payments, and boosting payments for new antibiotics ^73, 74^.

A further suggestion has been the creation of public benefit corporations as hybrids of for- and non-profit approaches ^75^. Similar to other public utilities, such corporations would combine operations for the social good with a moderate profit incentive and be able to access both public and private capital; core capital would be provided by charities and public sector entities, and shares would be sold with the expectation of moderate profits. However, substantial pull incentives like market entry rewards would still be needed to maintain long-term viability. Finally, a fully non-profit model has been proposed, where the requirements for returns are lower and any profit resulting from the sale of antibiotics could be reinvested in continuing development ^76^. GARDP is an example of such a non-profit entity that aims to develop five new treatments by 2025 ^77, 78^. All of these proposals are aimed at simultaneously incentivising the development of novel antibiotics, and enabling access for those who need them.

Many of the economic solutions described above are challenging to implement because they do not address the basic paradox of section 3; namely that, despite the societal costs of untreatable resistant infections, there is currently no viable market for new antibiotics. In the absence of such a self-sustaining market, public funds are being mobilised to create and sustain a market that is artificial.

While it is likely that we will need a range of public and private solutions if we are to solve a challenge of this magnitude in a way that delivers results not just for the next five years but for decades to come, it is unlikely that trying to recreate a broken marketplace will achieve this goal. However, if we acknowledge that there is no market for antibiotics, and acknowledge that there are bold new economic proposals being developed, does that open new ways for us to work together on the science?

## 6) Traditional development approaches are tied to secrecy

It is important to note what is shared between all the proposed economic solutions to drug development: *secrecy*. Though early-stage data may be shared eventually, all downstream drug development is conducted out of the public eye such that intellectual property may be protected using patents; that intellectual property (IP) is leveraged to recoup financial investment (ideally with a significant profit margin). This has three effects: 1) the preclinical R&D is less effective because it is less collaborative; 2) there is a persistent danger of unnecessary duplication of effort and therefore increased cost; and 3) the discovery and development of a new antibiotic remains thought of only as a ‘private sector’ problem, rather than a challenge for us as a society and a consequent public good. If there is no market for antibiotics, and if traditional protection of intellectual property is not needed, then what if we abandoned secrecy altogether?

## 7) We advocate for an additional, open approach to antibiotic R&D

Given the significant problems caused by the requirement for return-on-investment in current thinking, we advocate removing the requirement for secrecy and IP protection. The alternative is a transparent drug development approach in which all data and ideas are shared, anyone may participate, and advances will not be protected by patents. Adopting such an ‘open source’ approach acknowledges its success in creating market-leading, robust software underpinning much of the world’s online productivity ^79^. There exist biomedical research projects benefiting from the open sharing of data and expertise in this way in the areas of chemical probe discovery ^80^, the physical sharing of drug libraries ^81^, and in drug discovery ^82, 83^. Such pilots have shown how open projects can democratize discovery and development by allowing active participation by highly qualified people (individuals through to crowds) who may not otherwise have an avenue to contribute ^84^. Interestingly, there are examples of valuable data freely shared from private sector programs only once the original program has been dropped (e.g., the sharing of Novartis and Achaogen data on the SPARK platform) ^54^. Naturally there is a difference in scale between such activities and what is needed for a fully-fledged drug development program that would impact the fight against AMR, but the principles involved are the same.

We propose that the necessary elements of the discovery and development of new antibiotics are performed in the public domain, where everyone has access to all the same information. ‘Ownership’ of the project (website content and the associated data/chemical structures) may be effected clearly through an appropriate licence, similar to that which already governs other open-source projects such as Wikipedia but adapted to include aspects not well covered by such licences, such as physical samples. The general routes by which the various stages of such a project could be operated and managed have previously been outlined ^85^. There exist solutions already for some of the potential hurdles and novelties involved in such an endeavour, for example possible means for production/distribution (e.g. exploiting existing infrastructure underpinning the generics industry) and stewardship (via a series sponsor or other agency that would be involved in clinical trials in the same way as for any other development project). There are many other potential challenges in establishing such a new approach, such as how to convince potential funders of the economic feasibility of the approach (see section 9), how to replace IP and patent outcomes as a mark of success in this field and how to recognise the contributions to such projects towards a researcher’s career progression.

An existing early-stage antibiotic discovery project has recently been initiated as an example of how the open-source approach might operate, and as a way to identify more clearly the obstacles to success ^86^. A key criterion is to develop an effective medicine that is affordable and made appropriately accessible yet which would be subject to the same international best practice around responsible use and stewardship. As we write this article, there is a marked increase of similar calls for a more open culture towards coronavirus research in order to accelerate the development of an affordable cure, and many of the same issues will apply ^87^.

It should be noted that the development of openly-available assets is not incompatible with recent stimulus proposals such as the PASTEUR Act and the AMR Action Fund, providing strong financial incentives for investment in such projects. A further interesting aspect of open projects is of note here. There is a significant risk with the deployment of push and pull incentives that, if they are unbalanced, assets could build up and languish if they are seen as insufficiently promising for private sector capital investment, potentially wasting the original public sector investment. If all assets are openly available, they may instead be taken on by any sponsor.

## 8) What are the risks of this approach?

Objections raised to an open way of working may centre on the fear that ‘people will take what you have and run with it, in secret’; this may disincentivize contributions (for fear of losing credit for those contributions) even though patient benefit could still arise. Such ‘taking and running’ is possible, but it is unlikely. Open projects typically self-assemble high quality teams of scientists and thereby develop both momentum and community buy-in, including from global pharma employees (as part of corporate social responsibility initiatives) who enjoy the freedom such projects offer. Since all results, and all future experiments, are in the public domain, it is a challenge for a competing group to scope out the non-intuitive space required for IP protection; this ‘scorched earth’ approach to public disclosure brings with it a significant responsibility for an open-source project to be completed once it has been started. More generally it is not clear why anyone would wish to invest in a traditional, closed project that is attempting to find a medicine similar to one arising from an open project that is committed to equitable pricing (particularly when the closed model has been shown to be financially unviable). For later manufacturing and distribution, competition between generics suppliers would exist for openly-derived medicines just as it does now. One might also envisage that licences to supply governments with such medicines can be auctioned, in the same way that companies supply major clients (e.g., WHO) with supplies of medicines for neglected tropical diseases.

Pharmaceutical firms interested in remaining in this area would be free to compete with any open approach, or to collaborate with such projects in return for agreements on any future profits that might be required to make the collaboration commercially palatable, or in return for benefits associated with public acclaim through corporate social responsibility ^88^. We have recently seen such acclaim for investment in public goods with the significant contributions from the private sector towards COVID-19 vaccines, for example in the Oxford-AstraZeneca project.

## 9) How could we fund open antibiotic R&D?

Open source research is not free. Contributions arise from all sectors (from seasoned professional researchers in the pharmaceutical industry through to cohorts of students and early career researchers), yet major funding would still be required to develop a new antibiotic and a sponsor would be required to take overall ownership.

Will there be innovative sustainable funding for open projects, to retain accessible technology platforms, training, and knowledge transfer to progress projects, particularly through the ‘valley of death’ and the more expensive later stages of development? The pursuit of a low-cost, effective new medicine will make open projects attractive to existing funding sources from governments ( *via* grants) ^89^, philanthropists ( *via* donations), and non-governmental organizations ( *via* honest brokership and in-kind coordination, e.g., Medicines for Malaria Venture, DNDi, GARD-P, and emerging charities). There are already existing advocates for ^90^, and agencies pursuing or supporting ^91, 92^, a public-good development model (without necessarily opening up the research). As mentioned above, the newer push and pull incentives designed to fix a broken market for antibiotics are compatible with an open source way of working, even if they were not explicitly developed with such an approach in mind. Thus, for example, early stages of discovery are eligible for funding through CARB-X, later stages through the AMR Action Fund, and post-approval financial support through a subscription model (of which the PASTEUR Act is an example).

The above mechanisms are broadly supported by all those involved in the development of new antibiotics, and it is important not to confuse such funding with “drug discovery by governments” which is not being proposed here. Yet beyond public or philanthropic funding, there is also a way to conduct the research in a company setting using existing regulatory data and/or market exclusivities specifically designed to increase innovation in drug development. A sponsor conducting open research resulting in an approved medicine can still benefit from existing, multi-year protections from generic competition in major jurisdictions (despite there being no patent protection), a strategy being tested by M4K Pharma, a for-profit company seeking a therapy for a rare, uniformly fatal paediatric brain tumour ^93^. The company is fully owned by a non-profit trust, to which any profits are gifted in pursuit of an affordable medicine. One can therefore combine the mission-oriented approach of a company with the pursuit of a new medicine using an inclusive, open research model. Notably, the United States offers a significant exclusivity extension for new antimicrobials that address serious or life-threatening infections. To this end has been founded Medicines for Infectious Disease (M4ID) Pharma which is intending to trial this model for pathogenic infections including antibiotics ^94^.

## 10) Conclusion

As there is no viable market for new antibiotics, we are free to open the process of drug development. Though there have been pilots of initiatives that demonstrate the associated benefits of open research, there is a significant difference in their scale versus the usual project size that results in the development of a new medicine. A combination of approaches will be needed to make fresh progress against AMR. We are seeing bold new economic ideas involving push/pull incentives and leveraging of market exclusivities; these can now be used to fund bold, open source research practices that will accelerate the science towards new medicines. Just as there is an urgent need for new antibiotics, there is a corresponding urgency in our need to test, at scale, bold new ideas that have not yet been properly explored.

## Data availability

No data are associated with this article.
