# Supplementary material for: There is no market for new antibiotics: this allows an open approach to research and development
Source: Wellcome Open Res. 2021 Jun 11;6:146. [Version 1] doi: 10.12688/wellcomeopenres.16847.1 (PMC8237369; doi:10.12688/wellcomeopenres.16847.1)
Supplement: Peer review history

# Peer review: Reviewer response for version 1

**DOI:** 10.21956/wellcomeopenres.18582.r44432

**Published:** 2021-07-12

**Authors:** Ursula Theuretzbacher

**Affiliations:** 1 Center for Anti-Infective Agents, Vienna, Austria

Antibiotics and resistance are major global concerns. Insufficient supply of new antibiotics which respond to the greatest medical need and the current economic antibacterial drug discovery ecosystem requires a rethinking of the traditional economic model.  The authors of the Open Letter by Klug et al suggest a potential alternative to the traditional patent- and profit-based R&D model. Traditional antibiotics work well in the low-resistance context and therefore, the size of the major markets in the US and the large countries in Europe is rather small. Small sales, slow uptake and investor hesitancy would cause more cases of bankruptcy of small companies in the future. The effectiveness of recreating artificial profit-based solutions by large public investment may not be sufficiently effective and not provide the antibiotics that are needed most worldwide. These realities have been well presented by the authors. They advocate for an open and collaborative approach to antibiotic R&D that could be integrated into current incentive schemes. Such a non-profit public health driven model that is publicly funded would clear the way to open source discovery. Pilot projects are described in this article. The open source non-profit model in drug discovery is not new but has not achieved momentum yet to be considered widely. A formidable challenge of an open-source model is the rejection of the non-profit thinking among industry including most SMEs but also academic researchers and most politicians. The idea of project ownership and protecting its possession is widely regarded as an irrefutable certainty without alternative. The opposition against new ideas can be massive and the proponents of a profit-driven pharma system have a strong influence on policy makers, investors and sponsors. These aspects would require wider public discussion and detailed plans including the need for an experienced result-driven management team, programme governance, sustainable funding, and collaboration to achieve a critical mass. The suggested open-source model presents certainly a stimulating alternative to the failing traditional economic models and should serve as a basis for more detailed analysis for future implementation.

 Some minor comments that may be considered by the authors:

- The current COVID19 pandemic may not be the best example for a public R&D project as it is an example of a combination of high public investment and support in most high-income countries and extreme blockbuster profit occurring and expected for the years to come. This also refers to the AstraZeneca collaboration beyond the self-defined acute phase of the pandemic.
- The Swedish model is the answer to severe shortage problems with some generic antibiotics. Sweden plans to use incentives to keep such old antibiotics on the market. The mechanisms of keeping antibiotics on the market may be different from pull mechanisms for new antibiotics but could serve as model for providing access to new antibiotics once they are available. The sentences in this paragraph are not easy to understand.
- The described public-private partnerships (CARB-X and IMI) are only resource-sharing in a very narrow sense with a high degree of secrecy outside the individual programme.
- It would be beyond the scope of this article to discuss the terms broken market or market failure. Therefore, it may be better to avoid these terms and the need to define them.

Does the article adequately reference differing views and opinions?

Yes

Are all factual statements correct, and are statements and arguments made adequately supported by citations?

Yes

Is the Open Letter written in accessible language?

Yes

Where applicable, are recommendations and next steps explained clearly for others to follow?

Yes

Is the rationale for the Open Letter provided in sufficient detail?

Yes

Reviewer Expertise:

Antibiotic R&D, pipeline analysis of antibiotics, funding strategies for antibiotic R&D

I confirm that I have read this submission and believe that I have an appropriate level of expertise to confirm that it is of an acceptable scientific standard.

# Peer review: Reviewer response for version 1

**DOI:** 10.21956/wellcomeopenres.18582.r44435

**Published:** 2021-06-25

**Authors:** Richard Alm

**Affiliations:** 1 CARB-X, Boston University, Boston, MA, USA

This provocative Open Letter highlights an area of significant medical concern - how to maintain a suitable pipeline of antibiotics that will continue to provide the foundation to modern medicine in an environment that is scientifically challenging and economically unrewarding.  The authors explain the significant progress that has been made over the last few years around push and pull incentives in an attempt to delay what many fear will be inevitable. They appropriately cite the recent coronavirus pandemic as an example of a more open and collaborative scientific effort to develop medical interventions.  However, one significant difference is the speed at which that crisis arrived which had a very large and immediate societal and economic impact making it impossible to ignore, whereas the onset of an AMR crisis is considerably more nuanced, on a different timescale, and has not received the same attention from the media or policymakers. 

 The authors promote an open discovery platform where data and strategies can be freely shared between interested parties.  Early-stage research is often funded through government funding or philanthropic organizations, and the authors cite some of the initiatives aimed at this work, with suggestions that funding for the proposed open discovery model could be made available through many of the established sources currently available.  However, successful development truly means the regulatory approval of new medicines, and there are less examples of these being developed via a public model, and the authors should provide some citations to support their statement around the “abundance of successful real-world models of public pharmaceutical development”.  Such an open-sourced development paradigm would certainly need to deliver an innovative and sustainable solution for the time, effort, and funds that are required to successfully trial and launch a new product.

 Whether the open-source model drives the scientific innovation that is badly needed in this space to truly identify and develop differentiated products is certainly thought-provoking and worthy of a debate, although the finer details around concept design and execution will be critical.

 Some minor points for consideration are:

- Citation 8 from PEW tracks agents in clinical development and the statement regarding 43 new therapeutic agents in clinical or preclinical development should be reworded for clarity as there are a greater number of novel entities in preclinical development.

 

- While it is true that several larger companies have exited research in the antibacterial arena, the reference citing the exit of GSK (Ref 33) actually refers to the potential sale of two marketed drugs rather than research, and GSK currently has active clinical development programs with several small molecules.

 

- The reference for the note added in proof for the eNPV model is required.

Does the article adequately reference differing views and opinions?

Yes

Are all factual statements correct, and are statements and arguments made adequately supported by citations?

Partly

Is the Open Letter written in accessible language?

Yes

Where applicable, are recommendations and next steps explained clearly for others to follow?

Yes

Is the rationale for the Open Letter provided in sufficient detail?

Yes

Reviewer Expertise:

Molecular biology, microbiology, genomics, epidemiology

I confirm that I have read this submission and believe that I have an appropriate level of expertise to confirm that it is of an acceptable scientific standard.

# Peer review: Reviewer response for version 1

**DOI:** 10.21956/wellcomeopenres.18582.r44433

**Published:** 2021-06-15

**Authors:** Terry Roemer

**Affiliations:** 1 Prokaryotics Inc, Union, NJ, USA

The Open Letter by Klug *et al. *entitled ‘There is no market for new antibiotics: this allows an open approach to research and development’ outlines a provocative potential solution to the multiple and massive challenges this industry has faced historically, and which seem unrelenting going forward. The authors provide, in the cogent and well-referenced description, how the commercial market for new antibiotics has collapsed as well as emerging innovative economic “push” and “pull” solutions to this problem by public and private stakeholders. However, to confront these challenges the authors propose that an ‘open-source’ solution to antibiotic development, which rejects the constraints posed by the broken antibiotic commercial market, is needed to solve the conundrum.

 Given the problems caused by the requirement for return-on-investment in the antibiotic space, an open and collaborative approach to drug discovery is advocated – one that removes the requirement for secrecy and intellectual property protection and promotes transparency as a force multiplier for drug development. Funding to support such an initiative could be available through the same sources available to private companies. Unlike the often-cited example of open-source principles driving the success of the internet, however, skepticism of its impact on antibiotic development seems warranted. How appropriate is this analogy to the ‘wet lab’ setting of drug development? Even if a parallel open-source development strategy were adopted to solve market forces, how many new issues are created? Issues of program governance, management, investigator incentives, timelines, and eventual sponsors of the commercial product remain. It is also difficult to imagine how the approach would be sufficiently scalable to successfully span the decade-plus years required through discovery, preclinical and clinical stages of development. Notwithstanding such concerns, the authors suggest an innovative potential solution to the issues we face and if applied even only to the discovery/early development space, it could be impactful.

Does the article adequately reference differing views and opinions?

Yes

Are all factual statements correct, and are statements and arguments made adequately supported by citations?

Yes

Is the Open Letter written in accessible language?

Yes

Where applicable, are recommendations and next steps explained clearly for others to follow?

Yes

Is the rationale for the Open Letter provided in sufficient detail?

Yes

Reviewer Expertise:

Microbial genetics, genomics, chemical biology and antibiotic R&D

I confirm that I have read this submission and believe that I have an appropriate level of expertise to confirm that it is of an acceptable scientific standard.
